# SerpinB3/B4 Abates Epithelial Cell-Derived CXCL8/IL-8 Expression in Chronic Rhinosinusitis with Nasal Polyps

**DOI:** 10.1155/2024/8553447

**Published:** 2024-03-21

**Authors:** Xiangting Bu, Ming Wang, Jing Yuan, Jing Song, Ge Luan, Jiaqi Yu, Yang Wang, Ying Li, Chengshuo Wang, Luo Zhang

**Affiliations:** ^1^Department of Otolaryngology, Head and Neck Surgery, Beijing TongRen Hospital, Capital Medical University, Beijing 100730, China; ^2^Beijing Key Laboratory of Nasal Diseases, Beijing Laboratory of Allergic Diseases, Beijing Institute of Otolaryngology, Beijing 100005, China; ^3^Department of Allergy, Beijing TongRen Hospital, Capital Medical University, Beijing 100730, China

## Abstract

**Background:**

Serine proteinase inhibitors, clade B, member 3 (SerpinB3) and B4 are highly similar in amino acid sequences and associated with inflammation regulation. We investigated SerpinB3 and B4 expression and their roles in chronic rhinosinusitis with nasal polyps (CRSwNP).

**Methods:**

The expression of SerpinB3 and B4 in nasal mucosa tissues, brush cells, and secretions from CRSwNP patients was measured, and their regulation by inflammatory cytokines were investigated. Their functions were also analyzed using air–liquid interface (ALI)-cultured primary human nasal epithelial cells (HNECs) and transcriptomic analysis.

**Results:**

Both SerpinB3 and B4 expression was higher in nasal mucosa, brush cells, and secretions from eosinophilic (E) CRSwNP and nonECRSwNP patients than in healthy controls. Immunofluorescence staining indicated that SerpinB3 and B4 were primarily expressed in epithelial cells and their expression was higher in CRSwNP patients. SerpinB3 and B4 expression was upregulated by interleukin-4 (IL-4), IL-5, IL-6, and IL-17a. Transcriptomic analysis identified differentially expressed genes (DEGs) in response to recombinant SerpinB3 and B4 stimulation. Both the DEGs of SerpinB3 and B4 were associated with disease genes of nasal polyps and inflammation in DisGeNET database. Pathway enrichment indicated that downregulated DEGs of SerpinB3 and B4 were both enriched in cytokine–cytokine receptor interactions, with CXCL8 as the hub gene in the protein–protein interaction networks. Furthermore, CXCL8/IL-8 expression was downregulated by recombinant SerpinB3 and B4 protein in ALI-cultured HNECs, and upregulated when knockdown of SerpinB3/B4.

**Conclusion:**

SerpinB3/B4 expression is upregulated in nasal mucosa of CRSwNP patients. SerpinB3/B4 may play an anti-inflammatory role in CRSwNP by inhibiting the expression of epithelial cell-derived CXCL8/IL-8.

## 1. Introduction

Chronic rhinosinusitis (CRS) is a heterogeneous inflammatory disease that develops in the mucosa of the nasal cavity and the paranasal sinus. CRS affects 8% and 10.9% of the total population of China and Europe, respectively, has a debilitating effect on the quality of life, and is associated with a heavy economic burden [1, 2]. CRS patients with nasal polyps (CRSwNP) commonly combine with lower airway inflammation such as asthma, and usually experience severe symptoms, require more surgeries, and have higher recurrence rates following surgery—important points to be considered during clinical treatment [3].

CRSwNP pathogenesis is associated with various inflammatory responses. CRSwNP can be divided into two subtypes based on the infiltration of eosinophils into the nasal mucosa: eosinophilic (E) CRSwNP and nonECRSwNP. ECRSwNP is characterized by type 2 (T2) inflammation, with high levels of cytokines such as interleukin 4 (IL-4), IL-5, and IL-13, while nonECRSwNP is characterized by type 1 (T1) and/or type 3 (T3) inflammation and is associated with increased levels of cytokines such as IFN-*γ* and/or IL-17 [4]. Proinflammatory cytokines such as IL-1*β*, IL-6, and IL-8 (CXCL8) are also associated with multiple subtypes of inflammation and play a vital role in CRSwNP [5].

Serine proteinase inhibitors (Serpins) are a superfamily of homologous proteins. Numerous studies have shown that clade B Serpins (SerpinBs) play important roles in various immune and inflammatory functions [6]. For example, SerpinB5 is expressed by epithelial cells and is involved in the immune response to ulcerative colitis [7]; while SerpinB10 is related to asthma by inhibiting the apoptosis of allergenic Th2 cells [8]. Moreover, SerpinB1 can restrict the production of neutrophil extracellular traps [9]. Recently, certain SerpinBs has been found to be associated with CRS. In a study on biomarkers related to inflammatory endotypes of CRS patients without nasal polyps (CRSsNP), SerpinB2 and SerpinB10 were found to be significantly increased in T2 CRSsNP, which can be regulated by IL-13 and STAT6 [10]. The expression of SerpinB2 was also found to be significantly increased in polyp tissue and exosomes in CRSwNP [11]. However, whether other SerpinBs are associated with CRS are largely unknown.

Increased expression of SerpinB3 and B4 have been found in some inflammatory conditions, such as asthma, atopic dermatitis (AD) and psoriasis, and tuberculosis [12]. In psoriatic skin lesions, increased SerpinB3 and B4 expression facilitate a feedforward mechanism to modulate immune response through Pso p27 [13]. In asthma and AD patients, the induction of SerpinB3 and B4 can be caused by elevated Th2 cytokines IL-4 and IL-13 [14, 15]. As CRSwNP is also a type 2 dominated inflammation, little is known on the relationship between SerpinB3/B4 and CRSwNP. Since human SerpinB3 and B4 are evolutionarily homologous that share 95% and 92% nucleotide and amino acid sequence identity, their regulation and function were often been discussed together [12]. The present study aimed to investigate the expression, regulation, and function of SerpinB3/B4 in the nasal mucosa of patients with CRSwNP.

## 2. Materials and Methods

### 2.1. Subjects and Specimens

This study was conducted at Beijing Tongren Hospital from March 2019 to May 2022. A total of 97 participants, including 36 patients with ECRSwNP, 30 patients with nonECRSwNP, and 31 healthy controls, were enrolled into the study at Beijing TongRen Hospital. CRSwNP was diagnosed based on the European Position Paper on Rhinosinusitis and Nasal Polyps 2020 guidelines [16]. ECRSwNP and nonECRSwNP were defined in accordance with previous studies, and were based on whether or not the percentage of infiltrating eosinophils exceeded 27% of the total cells in nasal tissue [17]. Patients undergoing septoplasty due to anatomic variations but with no sinonasal diseases were selected as controls. Healthy volunteers were also included as controls for noninvasive sampling of brush cells and secretions. Specific inclusion/exclusion criteria for the volunteers of both groups are as follows:

The inclusion criteria for volunteers in control group and CRSwNP group include: (i) age 18–75 years; (ii) underwent surgical treatment of a deviated nasal septum or diagnosed as CRSwNP; and (iii) informed written consent.

The exclusion criteria for volunteers in control group and CRSwNP group include: (i) CRSwNP, antrochoanal polyps, fungal rhinosinusitis, cystic fibrosis, immunodeficiency, or primary ciliary dyskinesia; (ii) sinonasal fungal or viral infection; (iii) treat with antibiotics or oral corticosteroids in the month prior to inclusion; and (iv) treat with biologics in the 3 months prior to inclusion.

The study was approved by the Medical Ethics Committee of Beijing TongRen Hospital, Capital Medical University, and Beijing Institute of Otolaryngology. All patients enrolled into the study provided written informed consent. All of them in the control, ECRSwNP, and nonECRSwNP groups were matched by ethnicity and geographic location. Detailed demographic and clinical characteristics of the participants are presented in Table S1.

### 2.2. Nasal Sample Collection and Preparation Procedure

Otolaryngology specialists assessed the visual analog scale (VAS) scores for all patients with CRSwNP, and biopsies were obtained from nasal polyps in each CRSwNP patient. Nasal tissues of uncinate process from patients with nasal septum deviation were collected for comparison as controls. All biopsy samples were immediately immersed in 0.9% normal saline and transport with ice and processed as previously described [18].

Nasal secretions were obtained through a scissored postoperative sinus sponge pack Merocel (Medtronic Xomed, Jacksonville, FL, USA) as previously described [18]. The sponge was inserted into the middle meatus of each nostril parallel to the sagittal plane for 5 min. Next, 1 mL of 0.9% normal saline was added to the sponge for extraction of the secretion, after which all sponges were stored at 4°C for 2 hr. Later the sponges were centrifuged at 1,500 *g* for 15 min at 4°C. The supernatants were collected and stored in aliquots at −80°C until further analysis.

Brush cells were collected as previously described [19]. Briefly, a nasal brush (Copan, Italy) was inserted in the inferior turbinate of CRSwNP patients and control subjects by direct visual inspection under nasal endoscopy. The brush was pressed against the surface of the nasal mucosa and rotated for more than 10 full turns to acquire mucosal cells. After sampling, the brush was soaked with 1 mL TRIzol reagent (Thermo Fischer Scientific, MA, USA) in RNase-free collection tubes for RNA extraction.

### 2.3. RNA Isolation, Reverse Transcription, and Real-Time PCR

Total RNA was extracted from nasal polyps, nasal mucosa, brush cells, and cultured cells using TRIzol reagent (Thermo Fischer Scientific, MA, USA) as previously described [20]. Concentrations and quality of the RNA were estimated using the NanoDrop 2000 (Thermo Fischer Scientific), and single-strand cDNA was compounded by using PrimeScript™ RT Master Mix (TaKaRa Biotechnology Inc., Shiga, Japan). Finally, quantitative real-time PCR was performed using TB Green Premix Ex Taq™ II (TaKaRa Biotechnology Inc.) on an Applied Biosystems ViiA 7 Dx System (Applied Biosystems, CA, USA) using 12 *μ*L reactions (6 *μ*L of SmartChip TB Green Gene Expression Master Mix, 0.2 *μ*L ROX reference dye II, 0.4 *μ*L of 10 *μ*mol/L of each primer, and 5 *μ*L of cDNA) to assess mRNA levels in the samples. Glyceraldehyde-3-phosphate dehydrogenase (GAPDH) was used as the reference gene. mRNA expression was calculated as arbitrary units (AUs) using the following transformation: expression = 2^(−*Δ*ct)^ × 1,000 AUs. The primer sequences used in this study are presented in Table S2.

### 2.4. Western Blotting

Total protein was extracted from nasal tissues of ECRSwNP patients, nonECRSwNP patients, and healthy controls using RIPA lysate containing proteinase inhibitor cocktail, and the concentration of each sample was measured using a BCA protein assay kit (Beyotime, Shanghai, China) as previously described [21]. 30 *μ*g of the total protein was loaded onto 10% sodium dodecyl sulfate-polyacrylamide gels and the different proteins separated electrophoretically in 90 min. The separated protein bands were transferred to a nitrocellulose membrane (Millipore Corp., MA, USA) and blocked with 5% nonfat milk. After incubating with antibodies, the blots were visualized using enhanced chemiluminescence (Thermo Fischer Scientific), and the intensity of each band was quantified using the VersaDoc imaging system (Bio-Rad Laboratories, CA, USA). Blots were probed with anti-SerpinB3 (1 : 2,000, Abcam, MA, USA), anti-SerpinB4 (1 : 2,000, Santa Cruz, CA, USA), and anti-GAPDH (1 : 5,000, Abcam) at 4°C overnight and further immunoblotted with HRP-conjugated IgG antibody (1 : 5,000, Cell Signaling Technology, MA, USA) at room temperature for 60 min, developed with enhanced chemiluminescence substrate (Millipore, Darmstadt, Germany) and chemiluminescence detection by ChemiDocTM MP Imaging System (Bio-Rad, United Kingdom). Band density was quantitated using the Image Lab™ software Version 6.0.0 (Bio-Rad, United Kingdom).

### 2.5. Enzyme-Linked Immunosorbent Assay (ELISA)

Nasal secretions were collected as previously described [22]. Briefly, samples were collected from study participants using inflation sponges and soaked in 500 *μ*L of 0.9% sodium chloride solution at 4°C for 2 hr. The samples were processed in a high-speed centrifuge spinning at 1,500 × *g* for 15 min at 4°C and the secretions stored at −80°C. SerpinB3 (#P29508, RayBiotech, Norcross, GA, USA) and SerpinB4 (#P48594, RayBiotech) concentrations in the nasal secretions, and IL-8 (RAB0319, Millipore Corp., MA, USA) concentration in supernatants from the basal chamber of air–liquid interface (ALI) cultures were measured using a “sandwich” enzyme-linked immunosorbent assay (ELISA) technique according to the manufacturer's protocols.

### 2.6. Immunofluorescence Assay

To localize the expression of SerpinB3 and B4, nasal tissues from healthy controls and ECRSwNP and nonECRSwNP patients were performed for immunofluorescence analysis, as previously described [23]. Briefly, paraffinized tissue sections were deparaffinized, rehydrated, and processed using retrieval buffer (pH 6.0; Dako, Agilent, Santa Clara, CA) before blocking with 10% goat serum and incubating overnight at 4°C with primary antibodies and then with Alexa Fluor 488- or 594-conjugated secondary antibodies for 1 hr at room temperature in the dark. The slides were then mounted with Antifade reagent with DAPI. SerpinB3 antibody (1 : 500; ab154971, Abcam) and SerpinB4 antibody (1 : 500; sc-28384, Santa Cruz) were used as the primary antibodies. Isotype controls without primary antibody were also stained in nasal tissues from ECRSwNP and nonECRSwNP patients, and healthy controls. All the photos took by Olympus camera and processed by FV10-ASW Viewer software (Ver.4.2b) with same parameters and without adjustment post-acquisition.

### 2.7. Cell Culture and Stimulation

Human primary nasal epithelial cells (HNECs) were isolated from nasal polyps obtained from patients with CRSwNP who underwent elective endoscopic sinus surgery. Following enzymatic digestion, the isolated HNECs were subjected to ALI cell cultivation, as described by Wang et al. [21] After 2 weeks, 20–100 ng/mL of inflammatory cytokines (IL-6, IL-1*β*, TGF-*β*1, IL-4, IL-5, IL-13, IFN-*γ* IL-17a, and IL-8) and 1 *μ*g/mL of recombinant SerpinB3 (6528-PI, R&D Systems, Minneapolis, MN, USA) or SerpinB4 (6437-PI, R&D Systems) proteins were separately added to the culture solution for 24 hr. Cells were then collected and analyzed by RNA sequencing.

### 2.8. Transcriptome Sequencing

RNA was extracted from ALI-cultured HNECs following stimulation with recombinant SerpinB3 and B4. Sequencing was performed as previously described [24]. The quantity and quality of RNA were measured using NanoDrop 2000 spectrophotometer (Thermo Fischer Scientific) and 2100 TapeStation Automated Electrophoresis System (Agilent Technologies Inc., CA, USA). Samples with RNA integrity values greater than 7.0 were selected and used to build the RNA sequencing library. Ribosomal RNA was removed and an RNA-seq library prepared for sequencing using the VAHTS Universal V8 RNA-seq Library Prep Kit (Vazyme Biotech, China) following the manufacturer's instructions. RNA sequencing was performed on the Illumina HiSeq platform and 150 bp paired-end reads generated.

### 2.9. Pathway Enrichment and Gene-Disease Association Analyses

Gene expression was quantified as fragments per kilobase of transcript per million mapped reads (FPKM). The DEseq2 package was used to identify genes that were differentially expressed between the groups, with *P* < 0.05 as the cutoff for significant differentially expressed genes (DEGs). The significant DEGs were loaded into Metascape (https://metascape.org/gp/index.html#/main/step1) for pathway enrichment and associated disease analysis. The parameters for Gene Ontology Biological Process (GO-BP) and KEGG pathway analyses were: minimum overlap = 3; *P* value cutoff = 0.05; minimum enrichment = 1.5. Diseases associated with the genes were identified using the DisGeNET database on the Metascape platform [25, 26].

### 2.10. Construction of Protein–Protein Interaction (PPI) Networks

The DEGs were uploaded to the STRING database (version 11.5; https://string-db.org/) for prediction of PPIs as previously described [27]. The filter condition for the construction of a PPI network had a combined score >0.15. Cytoscape (version 3.8.0), an open-source network visualization and analysis software, was used to visualize the PPIs [26]. Node connectivity degree is defined as the number of links incident upon a node and is an important measure of the importance of a protein within the PPI network. Topological analysis of PPIs included measurements of connectivity degree, with nodes having higher degrees being classified as hub genes within the PPI networks. The major regulated genes were selected based on the topological measures and cytokine-related pathways.

### 2.11. Small Interfering RNA (siRNA) Transfection

The sequence “CTTGTGAACGCAATCTATT” and “CTGCAACATATCATGTTGA” were targeted for simultaneously knockdown of SerpinB3 and B4, and designed siRNA was synthesized by RiboBio, China. HNECs were seeded at a density of 1.5 × 10^5^ cells per well on 24 well plates, and were incubated overnight at 37°C. Transfection of siRNA was performed with Lipofectamine RNAiMAX reagent (Invitrogen, USA) according to the manufacturer's protocol, as described by Liu et al. [28] Cells were harvested after 24 or 72 hr for real-time PCR or ELISA analysis.

### 2.12. Validation of the Effect of SerpinB3 and B4 on CXCL8/IL-8

After establishing the ALI cultures of HNECs, 1 *μ*g/mL of recombinant SerpinB3 (6528-PI, R&D Systems, Minneapolis, MN, USA) or SerpinB4 (6437-PI, R&D Systems) were separately added to ALI cultures. Cells and supernatant were harvested after 24 or 72 hr for real-time PCR or ELISA analysis to detect intracellular and extracellular CXCL8/IL-8 expression.

### 2.13. Statistical Analysis

Data were analyzed and graphs generated using GraphPad Prism V.8.0 software (GraphPad Software, CA, USA). Results are presented as mean ± standard deviation (SD). Descriptive statistics were used to present general information on the study participants, and the distribution of the data was assessed for normality. Mann–Whitney *U* test or Student's *t*-test was used to analyze differences between groups, depending on the normality of data distribution. Comparison of paired data, such as in vitro cultured cells, was analyzed using the Wilcoxon matched-pairs signed-rank test. Differences were considered statistically significant when *P* < 0.05.

## 3. Results

### 3.1. Upregulated SerpinB3 and B4 Expression in Patients with CRSwNP

**Figure 1:**
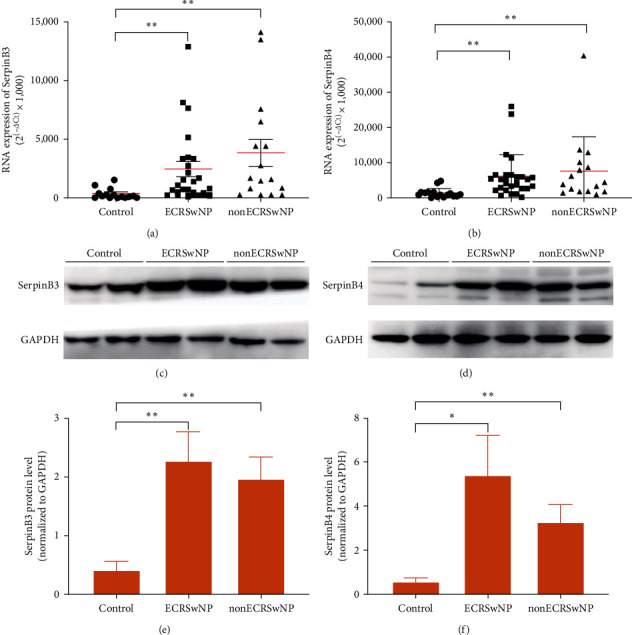
SerpinB3 and B4 expression in nasal tissues of patients with CRSwNP: (a, b) *SerpinB3* and *SerpinB4* mRNA levels in nasal tissues of ECRSwNP and nonECRSwNP patients measured by real-time PCR. *n* = 24 for ECRSwNP patients, *n* = 16 for nonECRSwNP patients, and *n* = 15 for healthy controls. RNA expression is measured from ABI ViiA™ 7 real-time PCR system and calculated as arbitrary units (2^(–*Δ*ct)^ × 1,000). Data are presented as mean ± SD and shown through Graphpad prism 8.0. GAPDH is used as the reference gene, (c, d) SerpinB3 and B4 protein levels in nasal tissues of nonECRSwNP and ECRSwNP patients and healthy controls measured by western blotting, and (e, f) intensities of western blot bands measured by image lab. Protein levels were normalized to those of GAPDH. *n* = 8 for ECRSwNP and nonECRSwNP patients and healthy controls.  ^*∗*^*P* < 0.05,  ^*∗∗*^*P* < 0.01,  ^*∗∗∗*^*P* < 0.001. CRSwNP, chronic rhinosinusitis with nasal polyps; ECRSwNP, eosinophilic CRSwNP; SerpinB, serine proteinase inhibitor, clade B; and GAPDH, glyceraldehyde 3-phosphate dehydrogenase.

Assessment of *SerpinB3* and *SerpinB4* mRNA expression showed that *SerpinB3* and *B4* expression were significantly higher in nasal tissues from patients with ECRSwNP and nonECRSwNP than in healthy controls (*P* < 0.01, respectively (Figures 1(a) and 1(b)). No significant difference was found in *SerpinB3*/*B4* expression levels between ECRSwNP and nonECRSwNP groups. SerpinB3 and B4 protein levels were evaluated using western blotting. The levels of both proteins were significantly higher in patients with ECRSwNP (*P* < 0.05) and nonECRSwNP (*P* < 0.01) than in healthy controls (Figure 1(c)–1(f)). These results indicate that SerpinB3 and B4 expression are elevated in ECRSwNP and nonECRSwNP patients both at mRNA and protein levels.

**Figure 2:**
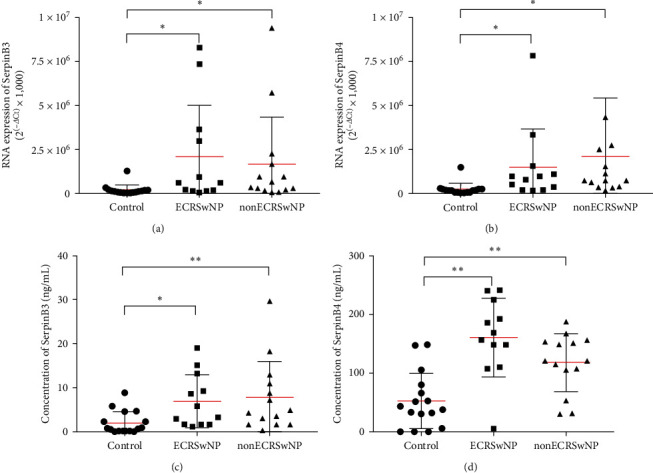
SerpinB3 and B4 expression in noninvasive samples from patients with CRSwNP: (a, b) *SerpinB3* and *SerpinB4* mRNA levels in nasal brush cells of ECRSwNP and nonECRSwNP patients measured by real-time PCR. *n* = 12 for ECRSwNP patients, *n* = 14 for nonECRSwNP patients, and *n* = 16 for controls. RNA expression is measured from ABI ViiA™ 7 real-time PCR system and calculated as arbitrary units (2^(–*Δ*ct)^ × 1,000). Data are presented as mean ± SD and shown through Graphpad prism 8.0. GAPDH is used as the reference gene and (c, d) SerpinB3 and B4 protein levels in nasal secretions of nonECRSwNP and ECRSwNP patients measured by ELISA. Protein content were measured by BioTek Epoch 2 Microplate spectrophotometer and produced by Epoch Gen5 software. Data are presented as mean ± SD and shown through Graphpad prism 8.0. *n* = 16 for ECRSwNP patients, nonECRSwNP patients, and healthy controls.  ^*∗*^*P* < 0.05,  ^*∗∗*^*P* < 0.01. CRSwNP, chronic rhinosinusitis with nasal polyps; ECRSwNP, eosinophilic CRSwNP; SerpinB, serine proteinase inhibitor, clade B; and GAPDH, Glyceraldehyde 3-phosphate dehydrogenase.

Noninvasive specimens, nasal brushing, and secretions from healthy controls and patients with ECRSwNP and nonECRSwNP were further analyzed using real-time PCR and ELISA to determine the expression of SerpinB3 and B4. *SerpinB3* and *B4* mRNA levels were significantly higher in brush cells collected from participants in the ECRSwNP (*P* < 0.05) and nonECRSwNP (*P* < 0.05) groups compared with those collected from participants in the control group. Additionally, there were no differences in mRNA levels between ECRSwNP and nonECRSwNP groups (Figures 2(a) and 2(b)). Similarly, the concentrations of SerpinB3 and B4 proteins in nasal secretions were significantly higher in patients with ECRSwNP (*P* < 0.05) and nonECRSwNP, compared to control group (*P* < 0.01, Figures 2(c) and 2(d)).

**Figure 3:**
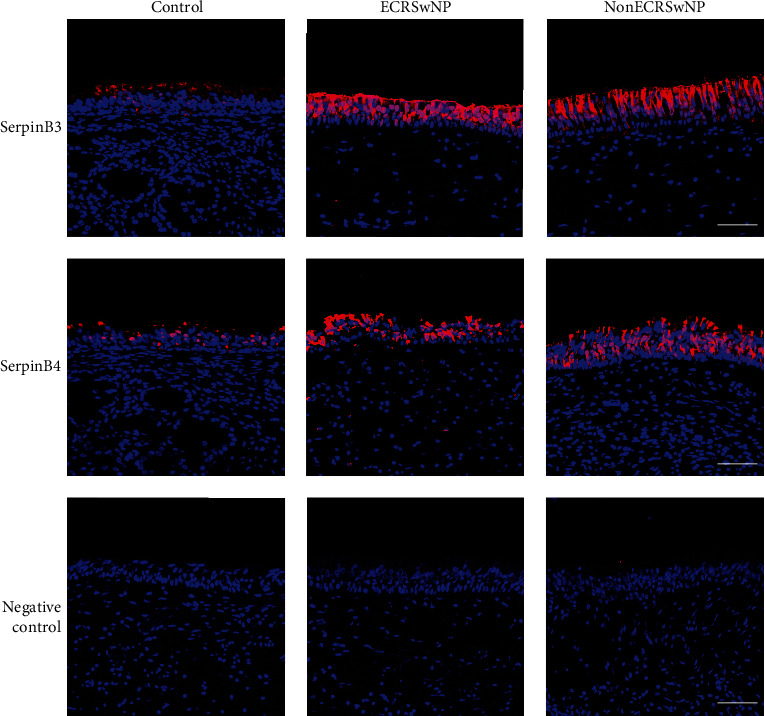
Localization of SerpinB3 and B4 in nasal tissues. Immunofluorescence staining of SerpinB3 and B4 (both in red) and negative control in nasal tissues collected from ECRSwNP and nonECRSwNP patients and healthy controls. Cell nuclei (blue) were visualized using DAPI counterstaining. Bars = 25 *μ*m. CRSwNP, chronic rhinosinusitis with nasal polyps; ECRSwNP, eosinophilic CRSwNP; SerpinB, serine proteinase inhibitor, clade B, and DAPI, 4′-6-Diamidino-2- phenylindole dihydrochloride.

Furthermore, we performed immunofluorescence staining of SerpinB3 and B4 on nasal tissues from participants in the ECRSwNP, nonECRSwNP, and control groups. Both SerpinB3 and B4 were predominantly expressed in the epithelial layer, while isotype control did not show any positive staining and the positive control CLU performed well (Figure 3 and Figure S3). Additionally, ECRSwNP and nonECRSwNP patients had relatively higher SerpinB3 and B4 levels compared with controls (Figure 3).

To elucidate the association between SerpinB3/B4 and disease severity, we conducted correlation analysis between SerpinB3 and B4 mRNA expression and VAS score of individual patients. Our findings revealed a positive correlation between SerpinB4 expression and VAS scores (*P* < 0.05; Figure S2), suggesting a potential link between elevated SerpinB4 expression and disease severity.

### 3.2. SerpinB3 and B4 are Regulated by Inflammatory Cytokines

**Figure 4:**
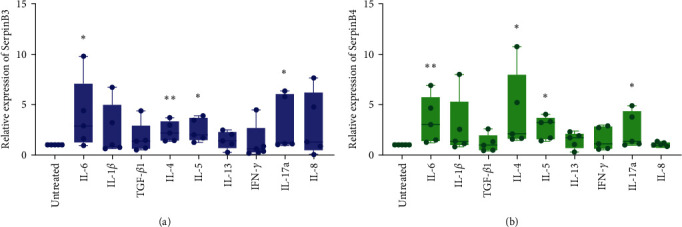
Regulation of SerpinB3 and B4 by cytokines: (a, b) *SerpinB3* and *SerpinB*4 expression are measured using real-time PCR 24 hr after stimulating ALI-cultured HNECs with IL-6, IL-1*β*, TGF-*β*1, IL-4, IL-5, IL-13, IFN-*γ*, IL-17a, and IL-8 (*n* = 5). RNA expression is measured from ABI ViiA™ 7 real-time PCR system and calculated as arbitrary units (2^(–*Δ*ct)^ × 1,000) and normalized according to untreated group. Data are presented as mean ± SD and shown through Graphpad prism 8.0. GAPDH is used as the reference gene.  ^*∗*^*P* < 0.05,  ^*∗∗*^*P* < 0.01. SerpinB, serine proteinase inhibitor, clade B; ALI, air–liquid interface; and HNECs, primary human nasal epithelial cells.

HNECs derived from CRSwNP patients were cultured *in vitro* under ALI conditions to mimic the native conditions of the airway epithelium. Cells were cultured for 2 weeks in ALI-condition and then incubated with IFN-*γ* (T1 cytokine), IL-4, IL-5, and IL-13 (T2 cytokines), IL-8 and IL-17a (T3 cytokines), TGF-*β*1 (epithelial remodeling cytokine), and IL-1*β* and IL-6 (proinflammatory cytokines) for 24 hr [29]. The effects of these cytokines on *SerpinB3 and B4* expression were measured using real-time PCR. *SerpinB3 and B4* expression was significantly upregulated by stimulation with IL-4, IL-5, IL-6, and IL-17a (*P* < 0.05, respectively; Figures 4(a) and 4(b)), but was not altered by cytokines IL-1*β*, TGF-*β*1, IFN-*γ*, IL-8, and IL-13.

### 3.3. Potential Function of Genes in Response to SerpinB3 and SerpinB4

**Figure 5:**
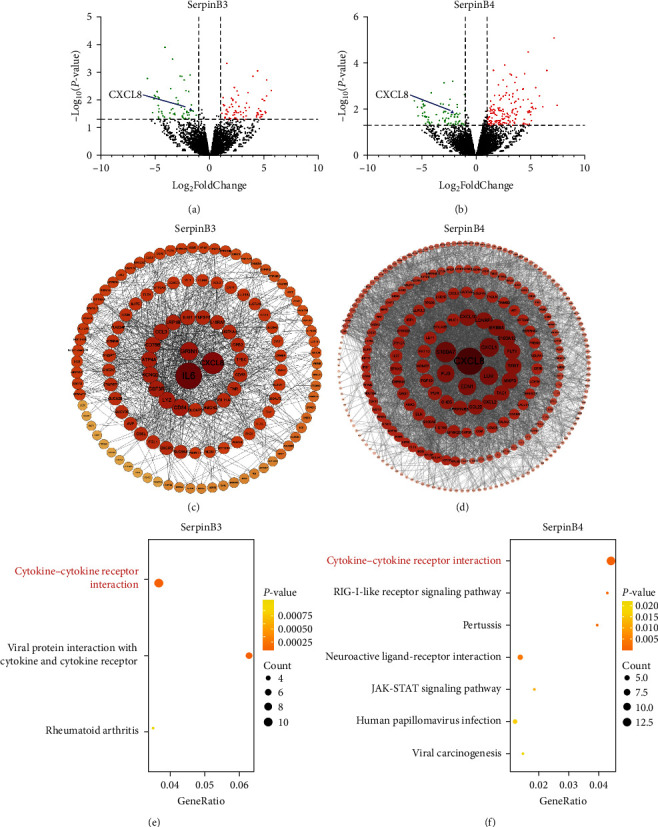
Genes and pathways in response to SerpinB3 and B4 stimulation. RNA sequencing analysis of ALI-cultured HNECs (*n* = 4) after 24 hr stimulation with recombinant SerpinB3 and B4, respectively: (a, b) volcano plots showing DEGs in response to SerpinB3 and B4. The red dots represent statistically upregulated DEGs while the green dots represent downregulated DEGs, (c, d) PPI networks of the 144 and 280 DEGs of SerpinB3 and B4, respectively. DEGs are arranged in circles by degree. The depth of the node color, font size, and node size reflect the degree of connection (darker colors, larger font size, and larger node size represent higher degrees). Top hub genes in the PPI networks are presented at the center of the circles, and (e, f) KEGG pathway enriched by the downregulated DEGs of SerpinB3 and B4, respectively. The number of genes enriched based on the enrichment terms is represented by the size of the node. PPI, protein–protein interaction; DEGs, differentially expressed genes; KEGG, kyoto encyclopedia of genes and genomes; SerpinB, serine proteinase inhibitor, clade B; ALI, air–liquid interface; and HNECs, primary human nasal epithelial cells.

ALI-cultured HNECs were stimulated with recombinant SerpinB3 and B4 proteins, respectively, and then performed RNA sequencing to investigate genes that potentially regulated by SerpinB3 and SerpinB4. A total of 144 DEGs were identified in SerpinB3-treated HNECs compared with the nonstimulated HNECs, including 78 upregulated and 66 downregulated genes (Figure 5(a)). Furthermore, 187 upregulated and 93 downregulated genes were identified in HNECs treated with SerpinB4 (Figure 5(b)). PPI networks were generated for the DEGs of SerpinB3 and SerpinB4, respectively. Based on the degree of genes in the PPI networks, IL-6 (degree, 84), CXCL8 (degree, 62), and GRIN1 (degree, 44) were identified as the top three hub genes that were potentially regulated by SerpinB3, whereas CXCL8 (degree, 140), S100A7 (degree, 106), and CXCL1 (degree, 98) were identified as the top three hub genes influenced by SerpinB4. Remarkably, CXCL8 is a common hub gene between the PPI networks of SerpinB3 and B4 (Figures 5(c) and 5(d)).

**Figure 6:**
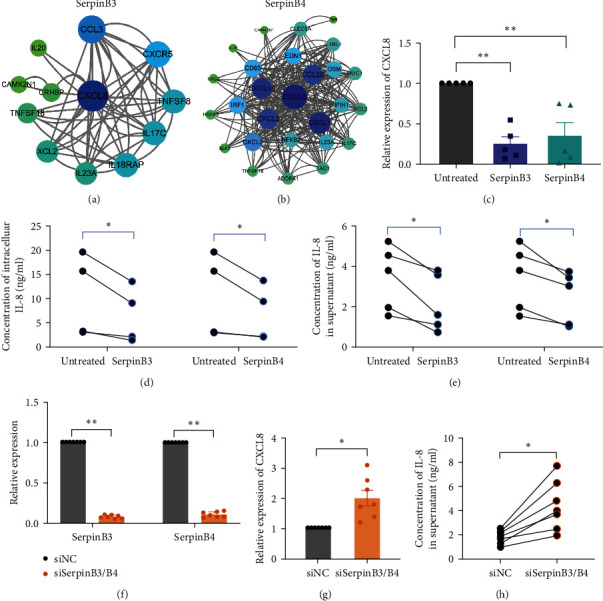
CXCL8 is downregulated by SerpinB3 and B4 in nasal epithelial cells: (a, b) PPI networks constructed by downregulated DEGs involved in the inflammatory pathway. The degree of genes is represented by the depth of the color and the size and font of the node, (c) CXCL8 expression validated by real-time PCR (*n* = 5), (d) the intracellular levels of IL-8 in cultured HNECs that treated with recombinant SerpinB3 and B4 (*n* = 4), (e) the concentration of IL-8 in supernatant of cultured HNECs that treated with recombinant SerpinB3 and B4 (*n* = 5), (f) expression of SerpinB3 and B4 after transfection with SerpinB3/B4 siRNA in HNECs (*n* = 7), detected by real-time PCR. SerpinB3/B4 siRNA was designed for simultaneously knockdown of SerpinB3 and B4. Black column represents siNC group, and orange column represent siSerpinB3/B4 group, (g) the gene expression of CXCL8 after transfected with SerpinB3/B4 siRNA in HNECs (*n* = 7), and (h) the production of IL-8 in supernatant of cultured HNECs (*n* = 7) that transfected with SerpinB3/B4 siRNA.  ^*∗*^*P* < 0.05,  ^*∗∗*^*P* < 0.01. PPI, protein–protein interaction; DEGs, differentially expressed genes; SerpinB, serine proteinase inhibitor, clade B; HNECs, primary human nasal epithelial cells, siNC, negative control siRNA, and siSerpinB3/B4, SerpinB3/B4 siRNA.

GO-BP and KEGG pathways enriched by those DEGs were analyzed to determine the possible downstream events in response to SerpinB3 and B4. As showed in Figures 5(e) and 5(f), Figure S4, DEGs were associated with inflammatory-related signaling pathways. For example, the cytokine–cytokine receptor interaction pathway was both enriched by DEGs of SerpinB3 and B4. Moreover, when subjected to GO-BP pathway enrichment, downregulated DEGs of SerpinB4 were related to positive regulation of cytokine production. Additionally, DEGs of both SerpinB3 and B4 were linked to neutrophil chemotaxis. The downregulation of specific DEGs, including CXCL8 and CCL3 by SerpinB3, and CXCL8, CXCL10, EDN1, CCL20, S100A12, CXCL1, CXCL3, CXCL2, and S100A8 by SerpinB4, was verified through real-time PCR (Figure 6(c)–6(e) and Figure S5). This validation indicates the inhibitory role of SerpinB3/B4 in impending neutrophil infiltration.

To further elucidate the molecular interactions, downregulated DEGs involved in the cytokine–cytokine receptor interaction pathway were further employed to construct PPI networks and visualized using Cytoscape software. Among the several cytokines downregulated by SerpinB3 and SerpinB4 in the PPI networks, CXCL8 was the central gene in both networks with highest connective degree (Figures 6(a) and 6(b)).

The DisGeNET database is a disease-related gene database that integrates information on gene-disease associations and variant-disease associations from several public data sources. Using the Metascape platform, we matched DEGs with the target genes of diseases in the DisGeNET database and found that both DEGs of SerpinB3 and SerpinB4 were associated with nasal polyps and inflammation (Figure S6(a) and S6(b)).

### 3.4. SerpinB3 and B4 Suppress CXCL8/IL-8 Expression in Nasal Epithelial Cells

We performed additional analysis to evaluate the expression of the hub gene, CXCL8, at both the RNA and protein levels in Figures 6(a) and 6(b). The results demonstrated a significant downregulation of CXCL8 upon stimulation with SerpinB3 and B4, compared to the untreated group, which was consistent with the sequencing results (*P* < 0.01; Figure 6(c)). Furthermore, there was a notable decrease in the concentration of intracellular IL-8 and the secretion level of IL-8 in the supernatant (*P* < 0.05, respectively; Figures 6(d) and 6(e)).

To further investigate the inhibitory effect of SerpinB3/B4 on CXCL8/IL-8 in HNECs, we used siRNA to simultaneously knock down the expression of SerpinB3 and B4 (*P* < 0.01; Figure 6(f)). Nucleotide alignment of SerpinB3 and B4 CDS showed high alignment score and the siRNA was designed targeting a common sequence in SerpinB3 and B4 CDS (Figure S1). The results demonstrated that the knockdown of SerpinB3 and B4 led to an upregulation of CXCL8 expression and an increase in IL-8 secretion in the supernatant (*P* < 0.05, respectively; Figures 6(g) and 6(h)).

## 4. Discussion

CRSwNP is characterized by persistent inflammation of the sinonasal mucosa, involving complex interactions among various immunological factors. SerpinBs have been implicated in providing cellular protection against proteases released from activated immune cells or lysosomes [30]. However, certain SerpinBs, including SerpinB2 and SerpinB10, have been shown to play a pathogenic role in lower airway inflammation [8, 31]. Therefore, it is crucial to investigate the expression and roles of SerpinBs under specific inflammatory conditions. Our study reveals a significant upregulation of SerpinB3/B4 expression in the nasal mucosa of CRSwNP patients compared to healthy controls. Furthermore, SerpinB3/B4 was found to suppress the expression of CXCL8/IL-8 derived from epithelial cells. Specifically, SerpinB4 was observed to inhibit the expression of neutrophil chemokines, including CXCL1, CXCL2, and CCL20, derived from epithelial cells. These findings suggest a potential anti-inflammatory role of SerpinB3/B4 in CRSwNP. To our knowledge, this is the first study to demonstrate the expression and functional significance of SerpinB3 and B4 in the context of CRSwNP.

We observed significantly higher SerpinB3/B4 expression in the nasal mucosa of patients with CRSwNP. Several studies have reported increased SerpinB3/B4 expression during inflammatory diseases such as allergic dermatitis [32], psoriasis [33], COPD [34], and asthma [35], indicating potentially important roles for SerpinB3/B4 in the regulation of inflammatory processes. As CRSwNP is a multifactorial and highly heterogeneous disease, we investigated the expression of SerpinB3/B4 in different CRSwNP subtypes, including ECRSwNP and nonECRSwNP. Comparable high expression of SerpinB3 and B4 was found between ECRSwNP and nonECRSwNP, and these results were supported by data from cultured nasal epithelial cells stimulated with inflammatory cytokines. SerpinB3/B4 expression can be upregulated by various inflammatory cytokines related to the pathogenesis of different endotypes of CRSwNP. For example, IL-4 and IL-5 which responsible for T2 inflammation, IL-17a which related to T2 inflammation, and the proinflammatory cytokine IL-6, are all contribute to the upregulation of SerpinB3/B4, consistent with results of previous studies [36]. Biological markers in nasal brushing and secretions provide valuable information on nasal pathophysiology. For example, CLC has previously been identified in nasal brush samples and can serve as a predictor of CRSwNP recurrence [37]. The inflammation-associated biomarker, cystatin SN, is present in nasal secretions and can be used to evaluate CRSwNP prognosis [18]. Our immunofluorescence staining indicates that SerpinB3 and B4 are mainly expressed in epithelial cells of the nasal mucosa, which may serve as noninvasive biomarkers for CRSwNP. Consistently, we found that the expression of SerpinB3/B4 are significantly increased in nasal brush cells and secretions from patients with CRSwNP, which is even more sensitive than that in nasal mucosa tissues.

The primary function of SerpinBs appears to be cellular protection against proteases released either from activated immune cells or from lysosomes [30]. Proteases that are highly expressed by neutrophils, mast cells, macrophages, and epithelial cells can lead to an increase in chemokines and proinflammatory cytokines that induce chemotaxis and leukocyte recruitment, and can therefore lead to persistent inflammation and lung infections [38]. SerpinB3 functions as a cathepsin L inhibitor, while SerpinB4 is an inhibitor of cathepsin G. Previous studies showed that both cathepsin L and G are associated with CRSwNP pathogenesis [39]. Besides as inhibitors of proteases, the function of SerpinB3 and B4 have not been investigated, especially in CRSwNP. Our findings demonstrate for the first time that SerpinB3/B4 can downregulate CXCL8/IL-8 expression in nasal epithelial cells. However, the mechanistic link between SerpinB3/B4 and CXCL8/IL-8 is still unclear. Interestingly, previous studies found that cathepsin L plays an important role in CXCL8/IL-8 processing, and cathepsin G can directly promote CXCL8 expression [40, 41]. Thus, one of the possible mechanisms that mediates CXCL8/IL-8 downregulation may be associated with the inhibitory role of Serpin B3/B4 on their targeted proteases.

CXCL8, a protein coding gene that encodes for IL-8, is a proinflammatory chemokine involved in promoting neutrophil chemotaxis and degranulation [42]. The expression of CXCL8/IL-8 is significantly upregulated during CRSwNP, and is also associated with disease severity. A recent study suggested that patients with elevated levels of CXCL8/IL-8 often have difficult-to-treat chronic rhinosinusitis [43]. Modulation of the overproduction of CXCL8/IL-8 may be one of the mechanisms through which inflammation is attenuated in many diseases. In the airway, p38 MAPK pathway is an important way in the regulation of CXCL8/IL-8 production [44]. Activation of p38 MAPK mediates epithelial CXCL8/IL-8 expression. It has been reported that SerpinB3 and B4 can inhibit the activation of p38 MAPK pathway by inhibiting the phosphorylation of p38 MAPK, which might be another mechanism in mediating the suppression of CXCL8/IL-8 [45, 46]. Overall, our findings suggest a protective role of SerpinB3/B4 in attenuating neutrophilic inflammation in CRSwNP.

While our findings demonstrated the inhibitory role of SerpinB3/B4 on CXCL8/IL-8 expression in nasal epithelial cells, it is important to note that CXCL8/IL-8 levels have been reported to be elevated in CRSwNP mucosa, particularly in cases with neutrophilic inflammation [47]. As CXCL8/IL-8 can be produced by various cell types and is subject to multiple upstream regulatory mechanisms. Apart from nasal epithelial cells, neutrophils, endothelial cells, and eosinophils are also known to produce CXCL8/IL-8 [48], which may contribute to the inconsistent observation of CXCL8/IL-8 levels originated from mucosa tissue and merely nasal epithelial cells in CRSwNP patients. In addition, CXCL8/IL-8 expression can be upregulated by inflammatory cytokines, such as IL-1 and TNF [49]. Therefore, further investigations are warranted to determine whether SerpinB3/B4 exert an inhibitory effect on CXCL8/IL-8 expression in other cell types.

Additionally, the cell location of SerpinB3 and B4 indicates that epithelial cells could be the most likely cells contacted and targeted by SerpinB3/B4. Previous studies have also suggested the roles of SerpinB3 and B4 on epithelial cells of lower airway [14, 35]. Our findings suggest that the impact of SerpinB3/B4 on inflammatory cells might be an indirect action mediated by CXCL8/IL-8. However, further study is needed to investigate whether SerpinB3/B4 have direct effect on inflammatory cells.

Our study however has some limitations. Firstly, the roles of SerpinB3 and B4 were investigated only using nasal epithelial cells. The effects of SerpinB3 and B4 on other models such as inflammatory cells and tissue explants need further exploration. Secondly, due to the high homology of SerpinB3 and B4, siRNAs commonly targeted SerpinB3 and B4 have been used in this study. Further separate investigations to discern the functional differences between SerpinB3 and B4 should be performed. Thirdly, both recurrent (5 of 24) and primary polyp samples were used to evaluate the expression of SerpinB3 and B4 in this study. As previous study showed that nonrecurrent and recurrent CRSwNPs have different types of inflammatory patterns [50], the recurrent rate should be considered a deviation factor.

In conclusion, our findings demonstrate that SerpinB3/B4 expression is upregulated in nasal mucosa of patients with CRSwNP and may function as noninvasive biomarkers for CRSwNP. SerpinB3/B4 may play an anti-inflammatory role in CRSwNP by inhibiting the expression of epithelial cell-derived CXCL8/IL-8.

## Data Availability

The data used in this study are shown as supplementary files.

## Abbreviations

CRSwNP: Chronic rhinosinusitis with nasal polyps
ECRSwNP: Eosinophilic chronic rhinosinusitis with nasal polyps
CRSsNP: Chronic rhinosinusitis without nasal polyps
CRS: Chronic rhinosinusitis
Serpins: Serine proteinase inhibitors
SerpinB3: Serine proteinase inhibitors, clade B, member 3
SerpinB4: Serine proteinase inhibitors, clade B, member 4
ALI: Air–liquid interface
HNECs: Primary human nasal epithelial cells
IL: Interleukin
DEGs: Differentially expressed genes
T: Type
COPD: Chronic obstructive pulmonary disease
GAPDH: Glyceraldehyde-3-phosphate dehydrogenase
AUs: Arbitrary units
FPKM: Fragments per kilobase of transcript per million mapped reads
GO-BP: Gene Ontology Biological Process
PPI: Protein–protein interaction
siRNA: Small interfering RNA.

## Supplementary Materials

Supplementary Materials Table S1: demographic characteristics of the study participants. Table S2: primers used for real-time PCR. Figure S1: nucleotide alignment of SerpinB3 and B4 CDS. Figure S2: correlation analysis of VAS score with SerpinB3 and B4 expression. This figure illustrates the correlation analysis between VAS scores and the mRNA expression levels of SerpinB3 (a) and SerpinB4 (b). SerpinB, serine proteinase inhibitor, Clade B; VAS, Visual Analog Scale. Figure S3: positive control for immunofluorescence staining. Immunofluorescence staining of antimicrobial protein CLU (in red) in nasal tissues collected from control subjects, ECRSwNP, and nonECRSwNP patients. Cell nuclei (blue) were visualised using DAPI counterstaining. CRSwNP, chronic rhinosinusitis with nasal polyps; ECRSwNP, eosinophilic CRSwNP; SerpinB, serine proteinase inhibitor, clade B; DAPI, 4′-6-Diamidino-2-phenylindole dihydrochloride. Figure S4: biological processes of Gene Ontology enriched by downregulated DEGs of SerpinB3 and B4. The *x*-axis represents GeneRatio. The number of genes enriched based on the enrichment term is represented by the size of the node. DEGs, differentially expressed genes; SerpinB, serine proteinase inhibitor, clade B. Figure S5: regulation of neutrophil chemokines by SerpinB3 and B4. Figure S6: diseases associated with DEGs of SerpinB3 and B4. DEGs of SerpinB3 and B4 were matched with target genes of diseases in the DisGeNET database on the Metascape platform (https://metascape.org/gp/index.html#/main/step1). The top 20 diseases are displayed in bar charts. DEGs, differentially expressed genes; SerpinB, serine proteinase inhibitor, clade B.

## References

[1] Shi J. B., Fu Q. L., Zhang H. (2015). Epidemiology of chronic rhinosinusitis: results from a cross-sectional survey in seven Chinese cities. *Allergy*.

[2] Hastan D., Fokkens W. J., Bachert C. (2011). Chronic rhinosinusitis in Europe–an underestimated disease. A GA^2^LEN study. *Allergy*.

[3] Promsopa C., Kansara S., Citardi M. J., Fakhri S., Porter P., Luong A. (2016). Prevalence of confirmed asthma varies in chronic rhinosinusitis subtypes. *International Forum of Allergy & Rhinology*.

[4] Stevens W. W., Peters A. T., Tan B. K., Klingler A. I., Kato A. (2019). Associations between inflammatory endotypes and clinical presentations in chronic rhinosinusitis. *The Journal of Allergy and Clinical Immunology. In Practice*.

[5] Bachert C., Akdis C. A. (2016). Phenotypes and emerging endotypes of chronic rhinosinusitis. *The Journal of Allergy and Clinical Immunology. In Practice*.

[6] Heit C., Jackson B. C., McAndrews M. (2013). Update of the human and mouse *SERPIN* gene superfamily. *Human Genomics*.

[7] Planell N., Lozano J. J., Mora-Buch R. (2013). Transcriptional analysis of the intestinal mucosa of patients with ulcerative colitis in remission reveals lasting epithelial cell alterations. *Gut*.

[8] Mo Y., Ye L., Cai H. (2021). SERPINB10 contributes to asthma by inhibiting the apoptosis of allergenic Th2 cells. *Respiratory Research*.

[9] Farley K., Stolley J. M., Zhao P., Cooley J., Remold-O"Donnell E. (2012). A SerpinB1 regulatory mechanism is essential for restricting neutrophil extracellular trap generation. *Journal of Immunology*.

[10] Klingler A. I., Stevens W. W., Tan B. K. (2021). Mechanisms and biomarkers of inflammatory endotypes in chronic rhinosinusitis without nasal polyps. *The Journal of Allergy and Clinical Immunology*.

[11] Mueller S. K., Nocera A. L., Dillon S. T., Libermann T. A., Wendler O., Bleier B. S. (2019). Tissue and exosomal serine protease inhibitors are significantly overexpressed in chronic rhinosinusitis with nasal polyps. *American Journal of Rhinology & Allergy*.

[12] Sun Y., Sheshadri N., Zong W.-X. (2017). SERPINB3 and B4: from biochemistry to biology. *Seminars in Cell & Developmental Biology*.

[13] Iversen O.-J., Lysvand H., Hagen L. (2011). The autoantigen Pso p27: a post-translational modification of SCCA molecules. *Autoimmunity*.

[14] Ray R., Choi M., Zhang Z., Silverman G. A., Askew D., Mukherjee A. B. (2005). Uteroglobin suppresses *SCCA* gene expression associated with allergic asthma. *Journal of Biological Chemistry*.

[15] Mitsuishi K., Nakamura T., Sakata Y. (2005). The squamous cell carcinoma antigens as relevant biomarkers of atopic dermatitis. *Clinical & Experimental Allergy*.

[16] Fokkens W. J., Lund V. J., Hopkins C. (2020). Isam European position paper on rhinosinusitis and nasal polyps 2020. *Rhinology*.

[17] Lou H., Meng Y., Piao Y., Wang C., Zhang L., Bachert C. (2015). Predictive significance of tissue eosinophilia for nasal polyp recurrence in the Chinese population. *American Journal of Rhinology & Allergy*.

[18] Wu D., Yan B., Wang Y., Wang C., Zhang L. (2021). Prognostic and pharmacologic value of cystatin SN for chronic rhinosinusitis with nasal polyps. *The Journal of Allergy and Clinical Immunology*.

[19] Chen H., Lou H., Wang Y., Cao F., Zhang L., Wang C. (2018). Comparison of the efficacy and mechanisms of intranasal budesonide, montelukast, and their combination in treatment of patients with seasonal allergic rhinitis. *International Forum of Allergy & Rhinology*.

[20] Bu X., Wang M., Luan G., Wang Y., Wang C., Zhang L. (2021). Integrated miRNA and mRNA expression profiling reveals dysregulated miRNA-mRNA regulatory networks in eosinophilic and non-eosinophilic chronic rhinosinusitis with nasal polyps. *International Forum of Allergy & Rhinology*.

[21] Wang M., Bu X., Fang G. (2021). Distinct expression of SARS-CoV-2 receptor ACE2 correlates with endotypes of chronic rhinosinusitis with nasal polyps. *Allergy*.

[22] Wang K., Chen L., Wang Y., Wang C., Zhang L. (2016). Sphenopalatine ganglion acupuncture improves nasal ventilation and modulates autonomic nervous activity in healthy volunteers: a randomized controlled study. *Scientific Reports*.

[23] Ma S., Xian M., Wang Y., Wang C., Zhang L. (2021). Budesonide repairs decreased barrier integrity of eosinophilic nasal polyp epithelial cells caused by PM_2.5_. *Clinical and Translational Allergy*.

[24] Wang M., Bu X., Luan G. (2020). Distinct type 2-high inflammation associated molecular signatures of chronic rhinosinusitis with nasal polyps with comorbid asthma. *Clinical and Translational Allergy*.

[25] Zhou Y., Zhou B., Pache L. (2019). Metascape provides a biologist-oriented resource for the analysis of systems-level datasets. *Nature Communications*.

[26] Shannon P., Markiel A., Ozier O. (2003). Cytoscape: a software environment for integrated models of biomolecular interaction networks. *Genome Research*.

[27] Hao Y., Wang B., Zhao J. (2022). Identification of gene biomarkers with expression profiles in patients with allergic rhinitis. *Allergy, Asthma & Clinical Immunology*.

[28] Liu T., Zhou Y. T., Wang L. Q. (2019). NOD-like receptor family, pyrin domain containing 3 (NLRP3) contributes to inflammation, pyroptosis, and mucin production in human airway epithelium on rhinovirus infection. *The Journal of Allergy and Clinical Immunology*.

[29] Tomassen P., Vandeplas G., Van Zele T. (2016). Inflammatory endotypes of chronic rhinosinusitis based on cluster analysis of biomarkers. *The Journal of Allergy and Clinical Immunology*.

[30] Kryvalap Y., Czyzyk J. (2022). The role of proteases and serpin protease inhibitors in *β*-cell biology and diabetes. *Biomolecules*.

[31] Poole A., Urbanek C., Eng C. (2014). Dissecting childhood asthma with nasal transcriptomics distinguishes subphenotypes of disease. *The Journal of Allergy and Clinical Immunology*.

[32] Sivaprasad U., Kinker K. G., Ericksen M. B. (2015). SERPINB3/B4 contributes to early inflammation and barrier dysfunction in an experimental murine model of atopic dermatitis. *Journal of Investigative Dermatology*.

[33] Ren C., Liu Q., Ma Y., Wang A., Yang Y., Wang D. (2020). TEAD4 transcriptional regulates SERPINB3/4 and affect crosstalk between keratinocytes and T cells in psoriasis. *Immunobiology*.

[34] Franciosi L., Postma D. S., van den Berge M. (2014). Susceptibility to COPD: differential proteomic profiling after acute smoking. *PLoS One*.

[35] Sivaprasad U., Askew D. J., Ericksen M. B. (2011). A nonredundant role for mouse Serpinb3a in the induction of mucus production in asthma. *The Journal of Allergy and Clinical Immunology*.

[36] Okawa T., Yamaguchi Y., Kou K. (2018). Serum levels of squamous cell carcinoma antigens 1 and 2 reflect disease severity and clinical type of atopic dermatitis in adult patients. *Allergology International*.

[37] Qi S., Yan B., Liu C., Wang C., Zhang L. (2020). Predictive significance of Charcot-Leyden crystal mRNA levels in nasal brushing for nasal polyp recurrence. *Rhinology*.

[38] Taggart C. C., Greene C. M., Carroll T. P., O’Neill S. J., McElvaney N. G. (2005). Elastolytic proteases: inflammation resolution and dysregulation in chronic infective lung disease. *American Journal of Respiratory and Critical Care Medicine*.

[39] Kao S. S.-T., Ramezanpour M., Bassiouni A., Wormald P.-J., Psaltis A. J., Vreugde S. (2019). The effect of neutrophil serine proteases on human nasal epithelial cell barrier function. *International Forum of Allergy & Rhinology*.

[40] Ohashi K., Naruto M., Nakaki T., Sano E. (2003). Identification of interleukin-8 converting enzyme as cathepsin L. *Biochimica et Biophysica Acta (BBA) - Proteins and Proteomics*.

[41] Guo J., Tu J., Hu Y. Y., Song G. X., Yin Z. Q. (2019). Cathepsin G cleaves and activates IL-36*γ* and promotes the inflammation of psoriasis. *Drug Design, Development and Therapy*.

[42] Kim D. H., Lim J. Y., Jang J. Y. (2023). Distinct subsets of innate lymphoid cells in nasal polyp. *Allergology International*.

[43] Liao B., Liu J.-X., Li Z.-Y. (2018). Multidimensional endotypes of chronic rhinosinusitis and their association with treatment outcomes. *Allergy*.

[44] Jundi K., Greene C. M. (2015). Transcription of Interleukin-8: how altered regulation can affect cystic fibrosis lung disease. *Biomolecules*.

[45] Moretto N., Bertolini S., Iadicicco C. (2012). Cigarette smoke and its component acrolein augment IL-8/CXCL8 mRNA stability via p38 MAPK/MK2 signaling in human pulmonary cells. *American Journal of Physiology. Lung Cellular and Molecular Physiology*.

[46] Murakami A., Suminami Y., Hirakawa H., Nawata S., Numa F., Kato H. (2001). Squamous cell carcinoma antigen suppresses radiation-induced cell death. *British Journal of Cancer*.

[47] Wang X., Sima Y., Zhao Y. (2023). Endotypes of chronic rhinosinusitis based on inflammatory and remodeling factors. *The Journal of Allergy and Clinical Immunology*.

[48] Matsushima K., Yang D., Oppenheim J. J. (2022). Interleukin-8: an evolving chemokine. *Cytokine*.

[49] Théâtre E., Bours V., Oury C. (2009). A P2X ion channel-triggered NF-*κ*B pathway enhances TNF-*α*-induced IL-8 expression in airway epithelial cells. *American Journal of Respiratory Cell and Molecular Biology*.

[50] Van Zele T., Holtappels G., Gevaert P., Bachert C. (2014). Differences in initial immunoprofiles between recurrent and nonrecurrent chronic rhinosinusitis with nasal polyps. *American Journal of Rhinology & Allergy*.

