# Supplementary material for: SerpinB3/B4 Abates Epithelial Cell-Derived CXCL8/IL-8 Expression in Chronic Rhinosinusitis with Nasal Polyps
Source: J Immunol Res. 2024 Mar 21;2024:8553447. doi: 10.1155/2024/8553447 (PMC10978078; doi:10.1155/2024/8553447)

**Supplementary Tables**

**Table S1.** Demographic characteristics of the study participants

| **Methodology** | **Control** | **ECRSwNP** | **nonECRSwNP** |
| --- | --- | --- | --- |
| **Real time-PCR of nasal tissues** | | | |
| Subjects | 15 | 24 | 16 |
| Gender, male (%) | 10 (66.7) | 18 (75) | 9 (56.3) |
| Age (years ± SD) | 49.3±12.8 | 43.6±8.05 | 47.3±12.9 |
| Atopy (%) | 5 (33.3) | 11 (45.8) | 6 (37.5) |
| Asthma (%) | 0 (0) | 13 (54.2)† | 4 (25)† |
| Recurrent rate (%) | - | 5 (20.8) | 1 (6.25) |
| **Mucosa eosinophils (%), median (IQR)** | NA | 46.0 (31.4-57.4)** | 12.3 (4-14.4) |
| **Mucosa neutrophils (%), median (IQR)** | NA | 8.8 (1.5-10.9) | 10.9(0.8-13.8) |
| **Mucosa plasma cells (%), median (IQR)** | NA | 14 .2(6.2-19.4) | 18.7 (9.5-25.7) |
| **Mucosa lymphocytes (%), median (IQR)** | NA | 32.0 (19.9-42)* | 56.8 (47.2-72.6) |
| **Real time-PCR and ELISA of non-invasive samples** | | | |
| Subjects | 16 | 12 | 14 |
| Gender, male (%) | 5 (31.3) | 8 (66.7) | 8 (57.1) |
| Age (years ± SD) | 27.44±6.06 | 43.25±9.17† | 45.86±13.16† |
| Atopy (%) | 1(6.3) | 8 (66.7)† | 4 (28.6)† |
| Asthma (%) | 0 | 8 (66.7)† | 3 (21.4)† |
| Recurrent rate (%) | - | 3 (25) | 0 |
| **Mucosa eosinophils (%), median (IQR)** | NA | 47.2 (33-60.8)** | 12.4 (4.4-18) |
| **Mucosa neutrophils (%), median (IQR)** | NA | 7.3 (1.5-12.4) | 10.1 (0.3-13.8) |
| **Mucosa plasma cells (%), median (IQR)** | NA | 16.2 (11.1-22.9) | 22.7 (9.3-29) |
| **Mucosa lymphocytes (%), median (IQR)** | NA | 32.1 (15-44.2)* | 66.9 (41-72.2) |

† *P* < 0.05, ECRSwNP or nonECRSwNP versus Control; * *P* < 0.05, ** *P* < 0.01, ECRSwNP versus non-ECRSwNP. ECRSwNP, eosinophilic chronic rhinosinusitis with nasal polyps; NA, not applicable; IQR, interquartile range.

**Table S2.** Primers used for real-time PCR

| **Primer** | **Sequence** |
| --- | --- |
| SerpinB3 | F:5'-TCGATGGTCTCCAGAAGCTTGA-3' |
|  | R:5'-TCCAGATAGCACGAGACCGC-3' |
| SerpinB4 | F:5'-GCCAAGGTCCTGGAAATACCA-3' |
|  | R:5'-TTCCATCAATTTCTCAGCAGTGA-3' |
| GAPDH | F:5'-CCTCTGACTTCAACAGCGACAC-3' |
|  | R:5'-TGGTCCAGGGGTCTTACTCC-3' |
| CXCL8 | F:5'-ACCGGAAGGAACCATCTCAC-3' |
|  | R:5'-GGCAAAACTGCACCTTCACAC-3' |

**Supplementary Figures**

**Figure S1. Nucleotide alignment of *Serpin*B3 and B4 CDS. A. Overview of *Serpin*B3 and B4 CDS via NCBI. This figure provides an overview of the nucleotide sequences for *Serpin*B3 and B4 coding regions sourced from NCBI. The horizontal bars are color-coded based on the alignment score. B. Detailed comparison of *Serpin*B3 and B4 CDS sequences. This panel offers a more in-depth comparison of the coding sequences of *Serpin*B3 and B4. Red bars represent 1-4 unmatched nucleobases, which identified through NCBI BLAST. siRNA of *Serpin*B3 and B4 also marked between 209-227 and 526-544. SerpinB, serine proteinase inhibitor, Clade B; CDS, Coding sequence.**

**
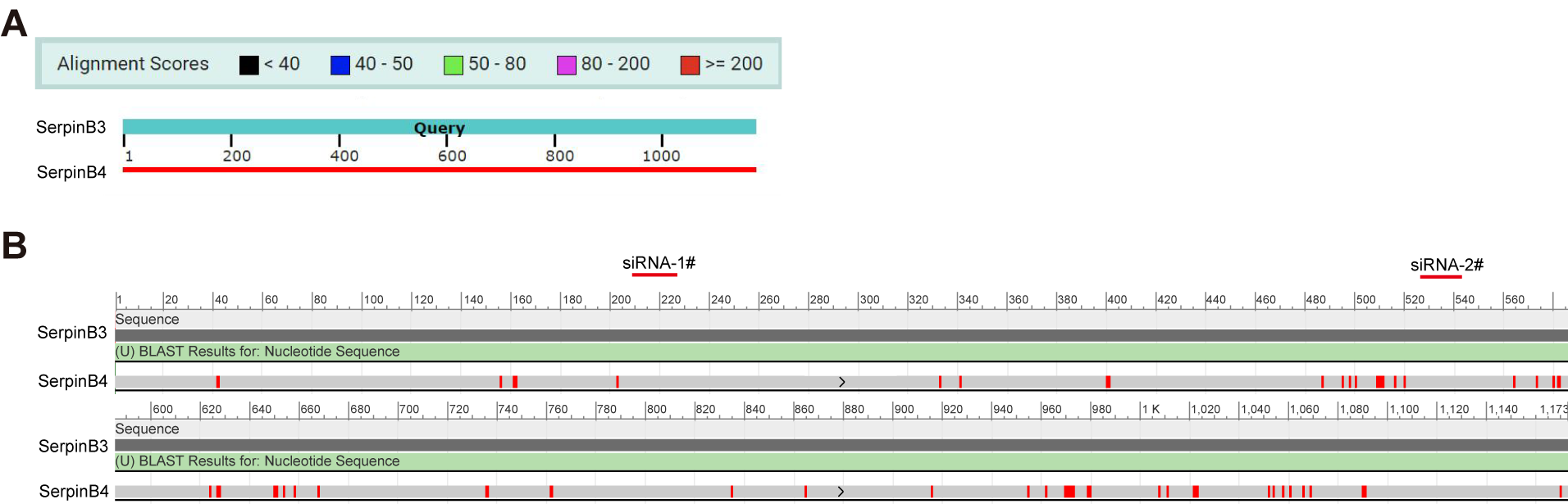
**

**Figure S2. Correlation analysis of VAS score with SerpinB3 and B4 expression. This figure illustrates the correlation analysis between VAS scores and the mRNA expression levels of SerpinB3 (A) and SerpinB4 (B). SerpinB,** serine proteinase inhibitor, Clade B; **VAS, Visual Analog Scale.**


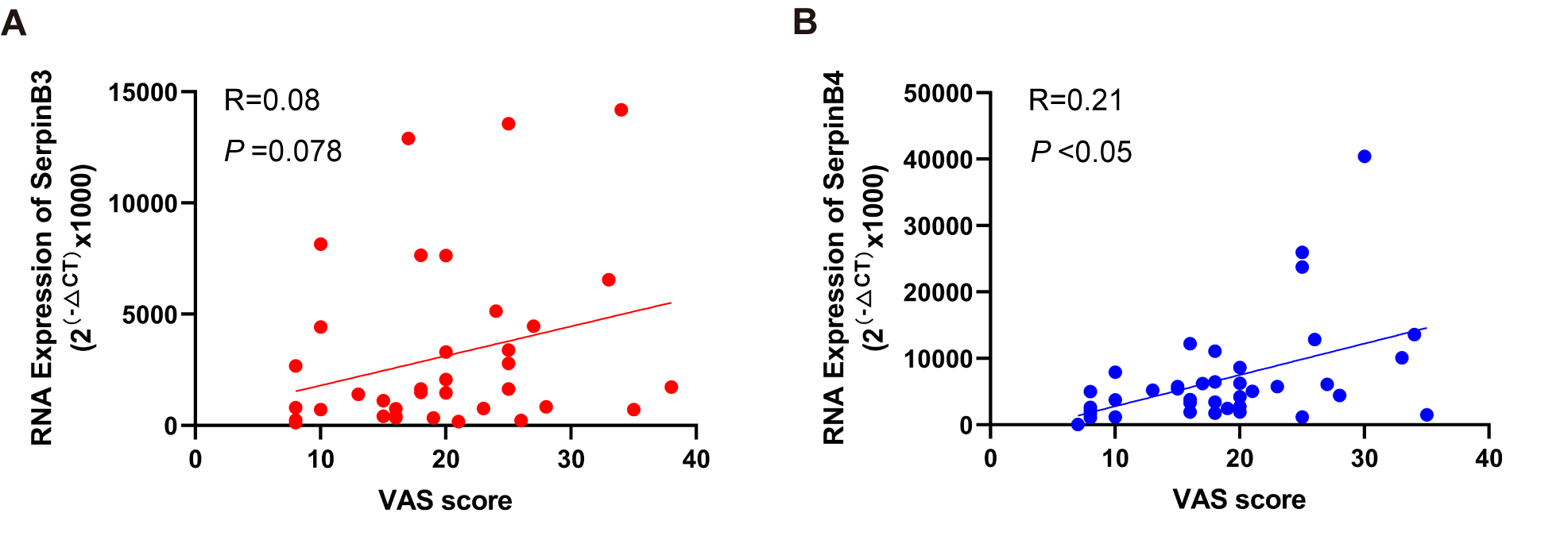


**Figure S3. Positive control for immunofluorescence staining. Immunofluorescence staining of antimicrobial protein CLU (red) in nasal tissues collected from Control subjects, ECRSwNP and nonECRSwNP patients. Cell nuclei (blue) were visualised using DAPI counterstaining. CRSwNP: chronic rhinosinusitis with nasal polyps; ECRSwNP: eosinophilic CRSwNP; SerpinB: Serine Proteinase Inhibitor, Clade B; DAPI, 4’-6-Diamidino-2- phenylindole dihydrochloride.**


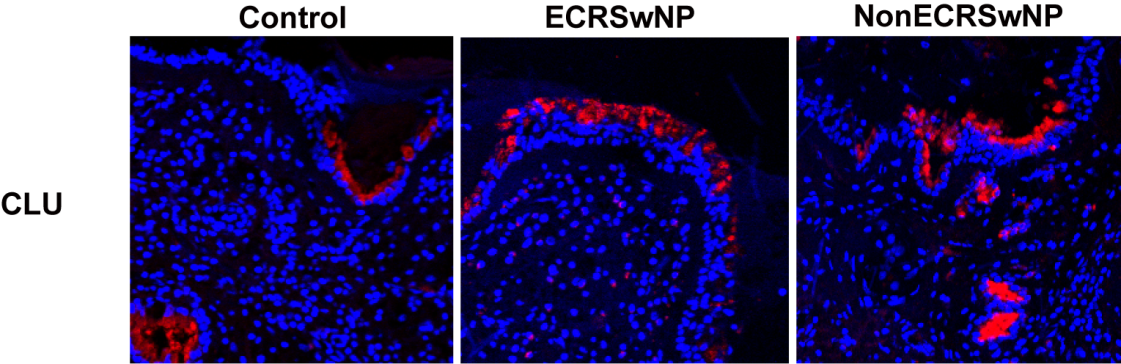


**Figure S4. Biological processes of Gene Ontology enriched by downregulated DEGs of** SerpinB3 **and B4. The x-axis represents GeneRatio. The number of genes enriched based on the enrichment term is represented by the size of the node. DEGs, differentially expressed genes; SerpinB, serine proteinase inhibitor, Clade B.**

**
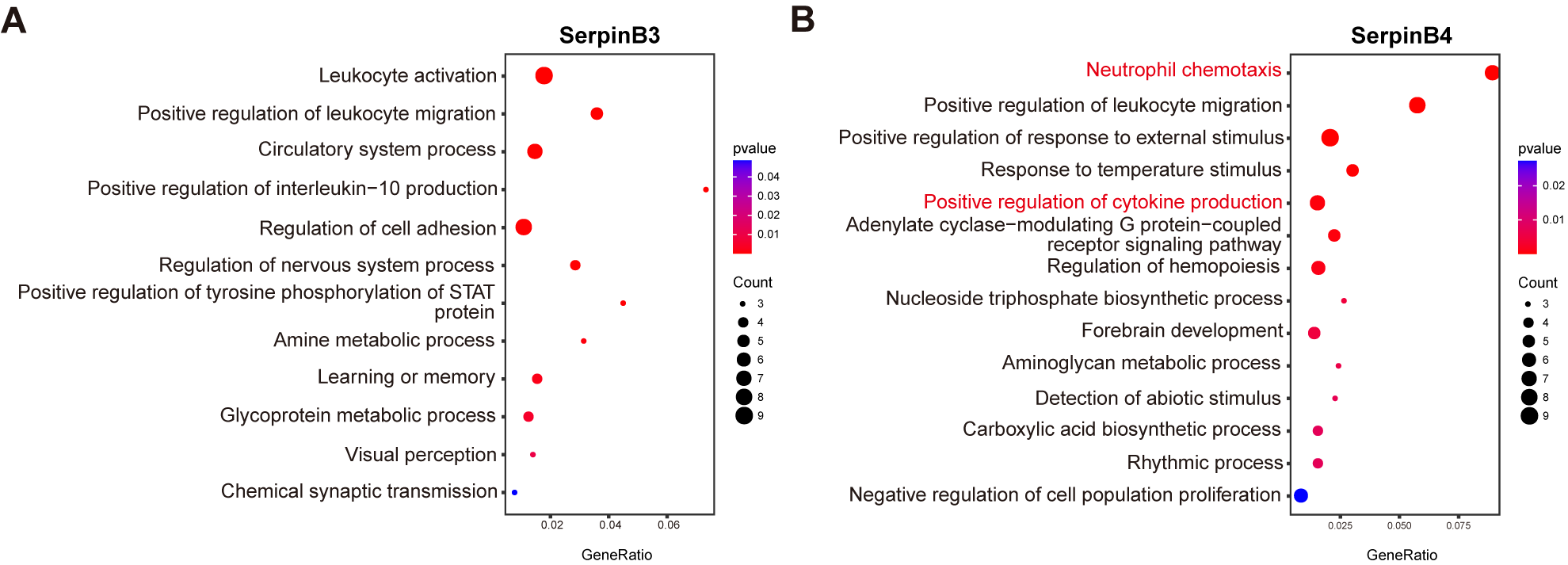
**

**Figure S5. Regulation of neutrophil chemokines by SerpinB3 and B4. A.** The FPKM values of neutrophil chemokines, including CXCL1, CXCL2, CXCL3, CXCL10, CCL20 (SerpinB4) and CCL3 (SerpinB3) were obtained from RNA sequencing analysis of ALI-cultured HNECs (n=4) following a 24 hour-stimulation with recombinant SerpinB3 and B4. B-G. The RNA expression of CXCL1, CXCL2, CXCL3, CXCL10, CCL20 and CCL3 was validated by real-time PCR (n=5). GAPDH served as the reference gene. * *P* < 0.05, ** *P* < 0.01. SerpinB: Serine Proteinase Inhibitor, Clade B; ALI, air-liquid interface; HNECs, primary human nasal epithelial cells; FPKM, fragments per kilobase of transcript per million mapped reads.


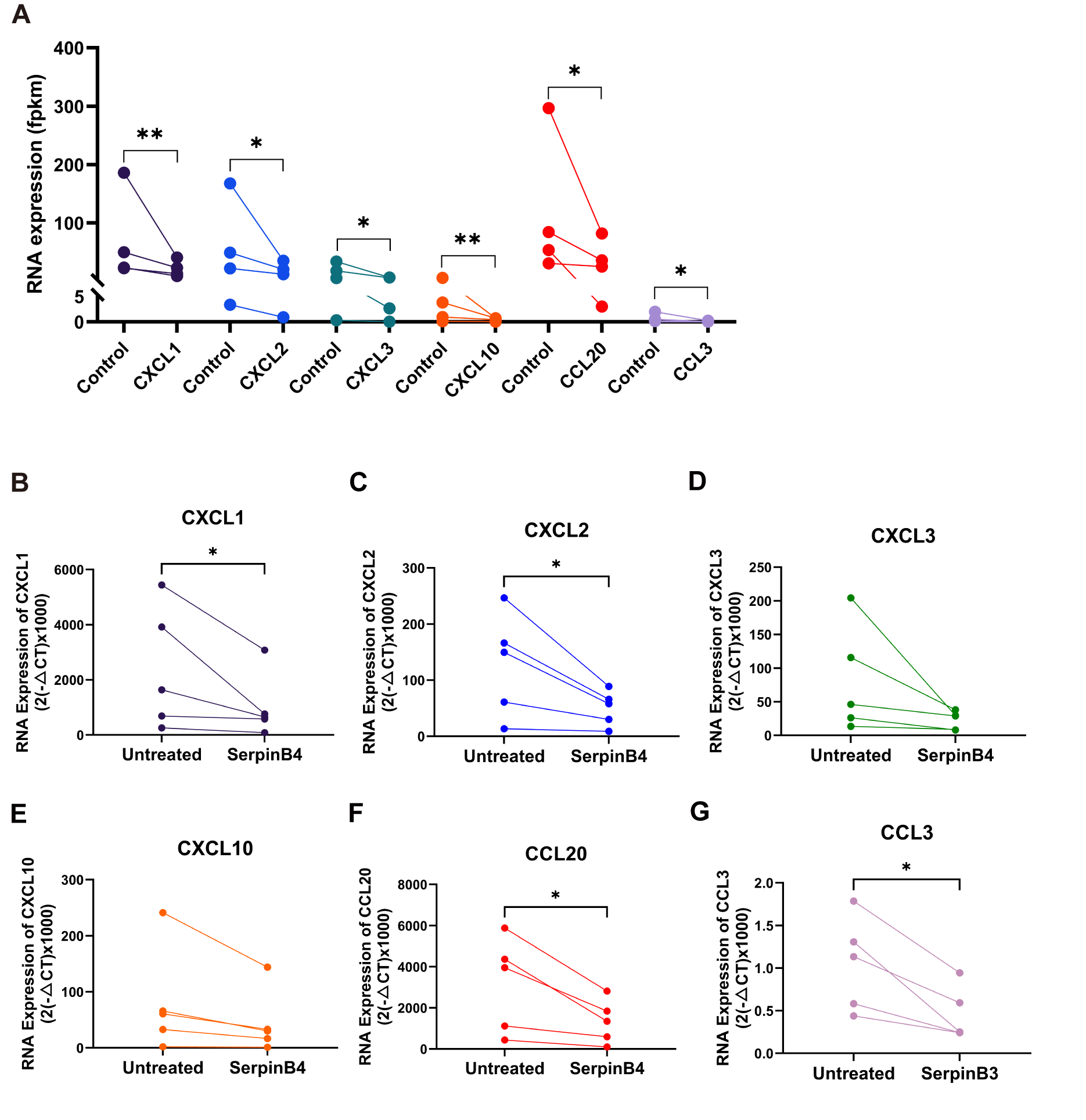


**Figure S6. Diseases associated with DEGs of** SerpinB3 **and B4.** DEGs of **SerpinB3 and B4 were matched with target genes of diseases in the DisGeNET** database on the Metascape platform (https://metascape.org/gp/index.html#/main/step1). **The top 20 diseases are displayed in bar charts. DEGs, differentially expressed genes; SerpinB, serine proteinase inhibitor, Clade B.**


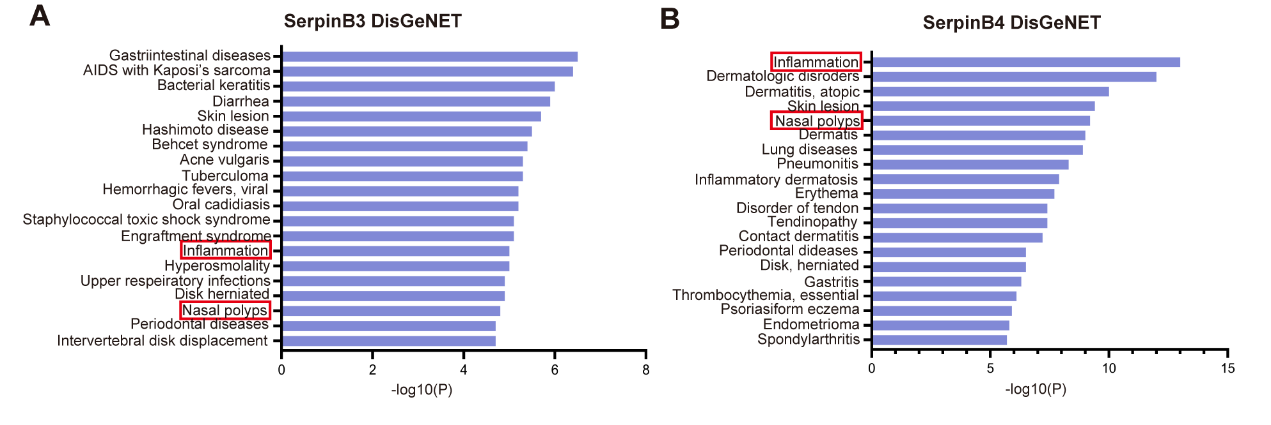

Supplement: Supplementary Materials — Table S1: demographic characteristics of the study participants. Table S2: primers used for real-time PCR. Figure S1: nucleotide alignment of SerpinB3 and B4 CDS. Figure S2: correlation analysis of VAS score with SerpinB3 and B4 expression. This figure illustrates the correlation analysis between VAS scores and the mRNA expression levels of SerpinB3 (a) and SerpinB4 (b). SerpinB, serine proteinase inhibitor, Clade B; VAS, Visual Analog Scale. Figure S3: positive control for immunofluorescence staining. Immunofluorescence staining of antimicrobial protein CLU (in red) in nasal tissues collected from control subjects, ECRSwNP, and nonECRSwNP patients. Cell nuclei (blue) were visualised using DAPI counterstaining. CRSwNP, chronic rhinosinusitis with nasal polyps; ECRSwNP, eosinophilic CRSwNP; SerpinB, serine proteinase inhibitor, clade B; DAPI, 4′-6-Diamidino-2-phenylindole dihydrochloride. Figure S4: biological processes of Gene Ontology enriched by downregulated DEGs of SerpinB3 and B4. The x-axis represents GeneRatio. The number of genes enriched based on the enrichment term is represented by the size of the node. DEGs, differentially expressed genes; SerpinB, serine proteinase inhibitor, clade B. Figure S5: regulation of neutrophil chemokines by SerpinB3 and B4. Figure S6: diseases associated with DEGs of SerpinB3 and B4. DEGs of SerpinB3 and B4 were matched with target genes of diseases in the DisGeNET database on the Metascape platform (https://metascape.org/gp/index.html#/main/step1). The top 20 diseases are displayed in bar charts. DEGs, differentially expressed genes; SerpinB, serine proteinase inhibitor, clade B. [file 8553447.f1.docx]
